# A Comprehensive Study of a Novel Explosively Hardened Pure Titanium Alloy for Medical Applications

**DOI:** 10.3390/ma16227188

**Published:** 2023-11-16

**Authors:** Michał Gloc, Sylwia Przybysz, Judyta Dulnik, Dorota Kołbuk, Marcin Wachowski, Robert Kosturek, Tomasz Ślęzak, Agnieszka Krawczyńska, Łukasz Ciupiński

**Affiliations:** 1 Faculty of Materials Science and Engineering, Warsaw University of Technology, 141 Woloska St., 02-507 Warsaw, Poland; agnieszka.krawczynska@pw.edu.pl (A.K.); lukasz.ciupinski@pw.edu.pl (Ł.C.); 2 Institute of High Pressure Physics, Polish Academy of Sciences (Unipress), 29/37 Sokolowska St., 01-142 Warsaw, Poland; sylwia@unipress.waw.pl; 3 Laboratory of Polymers and Biomaterials, Institute of Fundamental Technological Research, Polish Academy of Sciences, 4B Pawińskiego St., 02-106 Warsaw, Poland; jdulnik@ippt.pan.pl (J.D.); dkolbuk@ippt.pan.pl (D.K.); 4 Faculty of Mechanical Engineering, Military University of Technology, 2 Gen. S. Kaliskiego St., 00-908 Warsaw, Poland; marcin.wachowski@wat.edu.pl (M.W.); robert.kosturek@wat.edu.pl (R.K.); tomasz.slezak@wat.edu.pl (T.Ś.)

**Keywords:** explosive hardening, pure titanium, bioimplants, titanium alloys

## Abstract

Pure titanium is gaining increasing interest due to its potential use in dental and orthopedic applications. Due to its relatively weak mechanical parameters, a limited number of components manufactured from pure titanium are available on the market. In order to improve the mechanical parameters of pure titanium, manufacturers use alloys containing cytotoxic vanadium and aluminum. This paper presents unique explosive hardening technology that can be used to strengthen pure titanium parameters. The analysis confirms that explosive induced α-ω martensitic transformation and crystallographic anisotropy occurred due to the explosive pressure. The mechanical properties related to residual stresses are very nonuniform. The corrosion properties of the explosive hardened pure titanium test do not change significantly compared to nonhardened titanium. The biocompatibility of all the analyzed samples was confirmed in several tests. The morphology of bone cells does not depend on the titanium surface phase composition and crystallographic orientation.

## 1. Introduction

Pure titanium (CP Ti, ASTM—American Society for Testing and Materials F67 [1]) and titanium alloys (Ti6Al4V) (Ti6Al4V ELI, ASTM F136 [2]) are available on the market; both are used in medical applications due to their high corrosion resistance and resistance to the aggressive body [3].

Titanium is an allotropic metal with a hexagonal close-packed structure (HCP, α phase). In alloys, a body-centered cubic structure (BCC, β phases) and HCP structure are present [4]. The high specific strength, toughness and fatigue resistance required in medical applications depend on the β-phase [3].

The literature shows two significant problems with the commercially available Ti alloys: the low strength of CP Ti and the toxic/allergic effects of alloying elements made of the Ti6Al4V [3]. Cellular inflammatory reactions around bone-anchored percutaneous cochlear Ti alloy implants may occur [5]. Moreover, a patient with vanadium allergy may develop diffuse eczematous dermatitis and implant failure after receiving a vanadium-containing titanium alloy orthopedic implant in the left foot [6,7]. Reviews indicate several symptoms of allergy illness of patients with Ti alloy dental implants: facial inflammation, urticaria, pruritus, rash, dermatitis, facial eczema, welling in submental, labial sulcus, frank, pain and hyperaemia of soft tissues [8,9,10,11]. Titanium alloys may induce hypersensitivity in susceptible patients and could play a critical part in implant failure [8,12]. European data indicate that implant revision procedures are increasing [13,14]. Analysis of the tissue of patients after Ti alloy hip implantation showed the presence of macrophages and lesser T-lymphocytes and the absence of plasma cells and B-lymphocytes (a characteristic of delayed type IV hypersensitivity reaction) [15]. In Sweden, in 2000, 11,327 primary arthroplasties and 1573 revisions (12.2% revisions) were reported; in 2018, the respective numbers were 18,629 and 1863 (9.1%), respectively. Every revision generates pain for the patient, in addition to the costs of treatment. The literature warns against alternatives like cobalt–chromium (Co-Cr), which indicates suitable cytocompatibility parameters [16]. However, increased sensitization, allergic reaction and oral cavity inflammation may appear after dental implant mounting [17].

This publication aims to analyze the structure, mechanical properties and biocompatibility of pure-explosively hardened titanium (TE). Explosive deformation is currently one of the most modern innovative and unique methods of metal deformation used in practice. This technique allows materials with unique properties to be obtained, possessing both strength and resistance to corrosion. For example, explosion-hardened steel indicates the unique properties of nanomaterials, non-equilibrium and multi-layer materials [18,19]. It is worth emphasizing that the mechanism of the deformation process with an explosion and the method of the formation of complex structures has not been fully explained, and the results obtained so far are the subject of numerous discussions. Unique properties result from the deformation process’s speed, the significant speed of phase transformations and the rapid movement of dislocations in the material. Due to deformation, martensitic transformation might be generated, as in the case of other metals [19,20]. Under high pressure, the α phase in hexagonal close-packed (HCP) titanium transforms into the ω phase (simple hexagonal structure) [20]. It has been reported that α-ω martensitic transformation contributed to the enhancement of the selected mechanical properties. TE pure titanium is much more mechanically resistant than its undeformed counterpart, and is equal in mechanical strength to the alloys and additives currently used in commercially available titanium alloys. In this article, we describe titanium, which stands out in terms of its biocompatibility and durability, including its fatigue resistance, high strength and increased resistance to abrasion.

In this study, explosive deformation was used to enhance the mechanical properties of pure Ti grade 2. Firstly, the microstructure of an explosive deformation material was analyzed and compared to titanium before deformation (T) and Ti alloy (Ti6Al4V, TA) using WAXS. Subsequently, microhardness, tensile, compressive and corrosion tests were conducted. Appropriate to its application in medicine, the corrosion was analyzed. Finally, the cytotoxicity, bone cells morphology and synthesis of the selected ECM (extracellular matrix) proteins in contact with TE in comparison to T and TA were analyzed.

## 2. Material and Research Methodology

### 2.1. Sample Preparation

Titanium sheets of CP-Ti grade 2 (Wolften, Wrocław, Poland) were strengthened using the explosive method, which generated an optimal shock wave, and was described in the previous paper [19]. Briefly, the wave interacts directly with the tested material. The first stage involves cleaning the sheet’s contact surface with abrasive treatment. Then, the geometry of the sheets was adjusted through spark cutting. To evenly locate an explosive material on the plate surface, appropriate polymer frames were obtained using a 3D printing. Ammonal or emulsion explosives were used. After the explosion, the titanium sheets were cleaned. Then, the samples were cut using a wire-cut electric discharge machine (WEDM) AL-400S Accutex (Accutex, Taichung City, Taiwan) into 10 × 10 × 10 mm cubes. The developed titanium was indicated as “TE” (titanium after explosion). As controls, titanium alloy (Ti6Al4V), abbreviated to “TA”, and pure titanium (titanium grade 2), indicated as ”T”, were used.

### 2.2. WAXS Investigation

**Figure 1:**
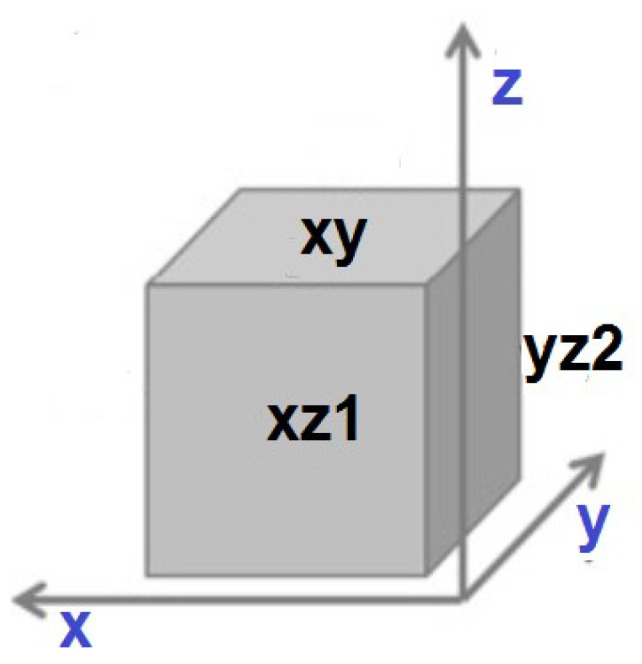
Scheme of the sample with labels associated to faces.

Wide angle X-ray scattering (WAXS) was applied for analysis of the phases and structure. WAXS measurements were performed using a Bruker D8 Discover diffractometer (Bruker, Billerica, MA, USA) with CuKa, λ = 0.154 nm radiation operated at a voltage of 40 kV and a current of 40 mA. All measurements were performed in rejection mode, using Goebel optics for beam formation: a 1 mm slit and Soller collimator. A highly sensitive Lynx Eye 1-D silicon strip detector was used. The range of diffraction angle, 2θ, was between 20° and 100°. The “empty” scan without a sample was subtracted and the default function of subtracting background was applied. WAXS was performed according to the procedure previously described [21]. Briefly, scans were taken at the center of the selected face of the cuboid: top (called XY direction) and from both sides (XZ1 and YZ2 (Figure 1).

### 2.3. Mechanical Properties—Low Cycle Fatigue Test

The low cycle fatigue test (LCF) was carried out on the specimens with a gauge width of 10 mm and length of 25 mm according to ISO standard 12106:2017 [22] in a total strain-controlled condition with asymmetry coefficient R = −1. The strain amplitudes of 0.35%, 0.4%, 0.45% and 0.5% were used. An axial clip extensometer with a gauge length of 25 mm was used to control the applied strain. The fatigue failure criteria were defined when the maximum tensile stress dropped by 20% below that at initial life. The fracture surfaces of the tensile and fatigue samples were explored using the Jeol JSM-6610 scanning electron microscope (JEOL, Tokyo, Japan).

### 2.4. Mechanical Properties—Residual Stresses

The measurements of residual stresses (RS) were conducted using the hole-drilling method (HDM). This procedure is standardized by ASTM Standard Test Method E 837 [23], and the practical procedure of implementation is described in detail in Tech Note TN-503 [24]. These measurements are used to determine the state of uniform residual stresses or their change with the thickness. In the presence of residual stresses, the realization of even a small hole in the material causes stress relaxation. This relaxation is related to the change in strain in the vicinity of the hole. Measuring the strain through strain gages allows the value and rotation of principal stress to be determined.

**Figure 2:**
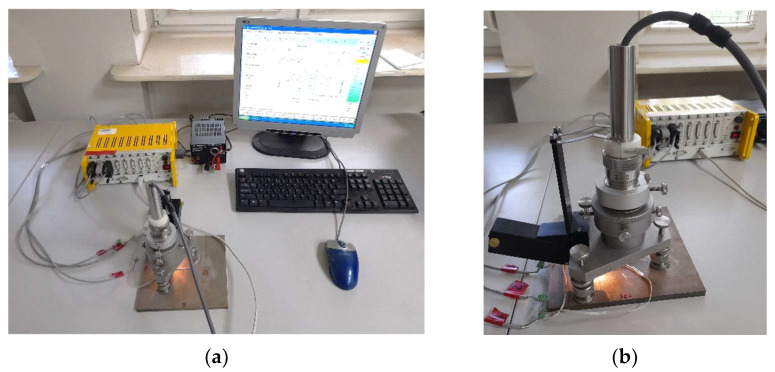
The overall view of the testing stand (**a**) and the milling guide of RS-200 while drilling the hole (**b**).

The procedure requires precise drilling of a hole in the center point of the strain gage rosette, which is the most important condition for obtaining the correct measurements. The Micro-Measurement 200 milling guide, which is powered by compressed air (Figure 2), was used for this drilling.

HDM requires the depth increment of a hole to be controlled during milling. This condition is ensured by RS-200, which enables the precise measurement of the hole depth increments with an accuracy of 0.01 mm and the measurement of hole diameter with an accuracy of 0.04 mm. The carbide milling cutters with a diameter of 1.6 mm were used during the tests and the blind holes were drilled at a depth of 2.0 mm.

**Figure 3:**
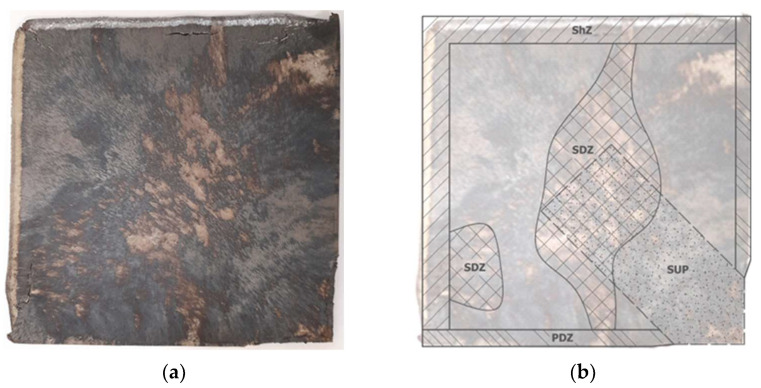
The view of the plate after EH (**a**) and the identified zones on its surface (**b**), namely: the shearing zone (ShZ), the plastic deformation zone (PDZ), the zone of significant changes (SCZ) and the zone in which there was a rigid support on the underside (SUP).

The measurements of residual stresses were conducted at the material Ti Grade 2 in the form of plates with dimensions of 175 × 175 × 8 mm; these took place in the delivery state and after explosive hardening (EH). The surface of the EH plate underwent many significant changes, as shown in Figure 3a and identified in detail in Figure 3b.

The place of detonation initiation was located in the lower right corner; therefore, there was a rigid support at the bottom (SUP). The propagating detonation wave caused a strong deformation of the plate edges, which were parallel to the propagation vector (PDZ). On the other hand, at the edges perpendicular to the direction of propagation, intense shear stresses occurred (ShZ). Moreover, in the central zone of the plate, severe surface changes appeared, with roughness that prevented the installation of strain gauge rosettes. SCZ is an area in which it was impossible to measure residual stresses.

### 2.5. Corrosion

Corrosion tests using direct current methods are one of the most universal corrosion measurement techniques due to their universality. The tests were performed using potentiostats equipped with an electrical system designed to maintain a constant potential between the tested electrode and the reference electrode and an accompaniment of a third platinum (auxiliary) electrode in the system. During the tests, anodic and cathodic polarization curves were obtained, which were used to determine the current and corrosion potential. Corrosion tests were carried out using an Autolab PGSTAT3 potentiostat (Metrohm AG, Herisau, Switzerland). Electrochemical direct current tests were performed using the potentio-dynamic method at a fixed bias voltage of 10 mV/s. Corrosion tests of the samples were carried out for samples in the initial state and after plastic deformation. The samples that were subjected to corrosion tests were tested in Ringer’s solution, the composition and characteristics of which are given in Table 1.

## 3. Cellular Studies

### 3.1. Materials

Dulbecco’s Modified Eagle Medium (DMEM), Foetal Bovine Serum (FBS), antibiotic (Penicillin-Streptomycin 10,000 U/mL), Trypsin EDTA (0.25%), Phosphate-Buffered Saline (PBS) pH 7.4, Presto Blue, NucBlue and ActinGreen were purchased from Thermo Fisher Scientific (Waltham, MA, USA). Mouse fibroblast L929 and human osteoblast MG63 were purchased from Sigma-Aldrich (ATTC), St. Louis, MO, USA.

In order to confirm the biocompatibility of the explosively hardened material made of pure titanium CP grade 2 (TE), in-vitro cell tests were performed: cytotoxicity tests on sample extracts and adherent cell cultures in direct contact with the material surface. Tests of the pure titanium materials after explosive deformation were performed with reference to the control material (T) at the initial state. In the case of the tests in direct contact, cells were cultured on the surface of a commercially available Ti6Al4V alloy. Before proceeding with the cellular tests, the material samples were ground with the abrasive paper of 80, 320, 600, 1200 and 2400 gradations, polished using the diamond paste of 1 µm and subjected to a two-step sterilization process. First, the samples were placed in an 80% ethyl alcohol solution for 30 min. Then, the samples were subjected to autoclaving at increased pressure and a temperature of 121 °C.

### 3.2. Cytotoxicity on Extracts

A cytotoxicity study on material extracts was performed using murine L929 fibroblast cells (Sigma-Aldrich (ATTC), St. Louis, MO, USA). A culture medium was used to obtain the extracts. The medium consisted of Dulbecco’s Modified Eagle Medium (DMEM), fetal bovine serum (FBS) and a mixture of antibiotics—penicillin and streptomycin. All samples were placed in a multi-well plate, immersed in culture medium and kept in an orbital shaker for 24 h and at 37 °C. Simultaneously with the start of the 24 h of obtaining extracts from the samples, cell cultures were started—10,000 cells per well in a 96-well plate. After 24 h, the medium in all of the cell culture wells was replaced with a material extract, or clean medium for the control. The culture plate was placed in the incubator for 24 h. After this time, the plate was tested for the viability of cells flattened at the bottom of the wells using the fluorescence assay PrestoBlue with extraction/emission 580/630 nm. (MultiscanGO Microplate Spectrophotometer, Thermo Scientific, Waltham, MA, USA)

### 3.3. Cell Adhesion and Proliferation Study in Direct Contact

Due to the fact that the obtained material should be used in a living organism and remains in contact with bone tissue, the MG63 cell line was selected for the study of the in-vitro cellular response in direct contact with the material. The optimal culture medium for the MG63 cell line consisted of the same medium composition as the one used with the L929 cell line, with additional supplementation in the form of glutamine.

For the purpose of this study, in direct contact with the material, cell culture was carried out for 3 and 5 days. The same cell seeding density was used for both time points, and seeding on both the top and side surfaces of the samples was used to evaluate the effect of the strain force direction on the cellular response. The samples were placed in the wells of 24-well plates and then seeded with cells with a density of 5000 cells per cm^2^ on all the tested surfaces (XY and XZ1). After 3 and 5 days, the culture plates were removed from the incubator and then fixed with a 3% formaldehyde solution and fluorescently stained. The cell nuclei were stained using NucBlue™ Fixed Cell ReadyProbes, which emit light with a maximum intensity at 460 nm with excitation at 360 nm. The actin skeleton was stained using ActinGreen™ 488 ReadyProbes, which emit light with a maximum intensity at 518 nm when illuminated with a light beam with a wavelength of 495 nm. Fluorescence microscopy images were taken for each culture in three magnifications: 10, 20 and 63 times (Leica DMI3000B, Wetzlar, Germany).

### 3.4. ECM Secretion Tests in Direct Contact

For the purpose of both types of tests, the top surfaces of all samples were seeded using the MG63 cell line at 2500 cells per cm^2^, and cultured for 7 days. The same culture medium was used as in the previously described tests that were performed with the MG63 cell line.

### 3.5. Direct Red 80—Collagen Deposit Detection

Sirius red is a dye for staining varying collagen types and available collagen and for the determination of its amount. Before staining, cells seeded on Ti samples were washed 2 times with PBS. Then, 1 mL of 0.1% *w*/*v* sirius red (Direct red 80, Merck, Rahway, NJ, USA) in picric acid (Chempur, Piekary Śląskie, Poland) was added to the wells of each 24 well-plate, and the samples were kept in this solution for 60 min in RT. Then, the solution was removed and the samples were washed 3 times with 5% *v*/*v* acetic acid. After this step, 1 mL of 0.1 M NaOH was added and kept in RT. After 30 min, 100 µL of each solution from each sample was transferred to a dedicated well of 96 well-plates, and the absorbance was measured at 460 nm using a Multiskan™ GO Microplate Spectrophotometer (Thermo Scientific™, Waltham, MA, USA).

### 3.6. Alizarin Red—Calcium Deposit Detection

The culture medium was removed from each well and the cells were washed gently with PBS 3 times. The cells were then fixed with 3% formaldehyde for 15 min. After removal of the formaldehyde solution, all wells were washed 3 times using demineralized water. Then, 1 mL of Alizarin red staining solution was added to each well and incubated for 30 min at RT with gentle stirring. After the dye was removed, the cells were washed 5 times with demineralized water and 200 μm of 0.5 M HCl was added to each well to release the stain. In order to increase the pH to the 4.1–4.5 range, a (x volume) od 1 M NaOH was added. The absorbance was measured at 430 nm using a Multiskan™ GO Microplate Spectrophotometer (Thermo Scientific™, Waltham, MA, USA).

## 4. Research Results

Titanium sheets of CP-Ti grade 2 were strengthened using the explosive method and their selected structure and mechanical properties were compered to titanium at the initial state (without deformation) and titanium alloy Ti6Al4V. Additionally, cytotoxicity analysis and cellular morphology were determined.

### 4.1. WAXS Results

**Figure 4:**
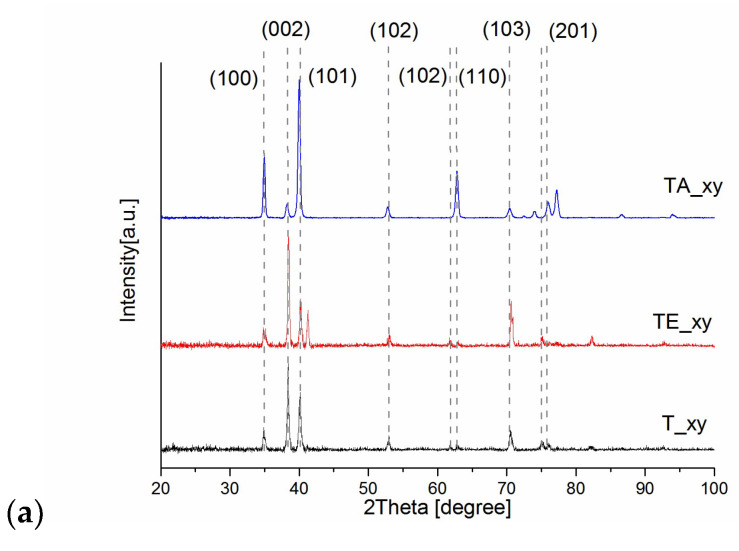

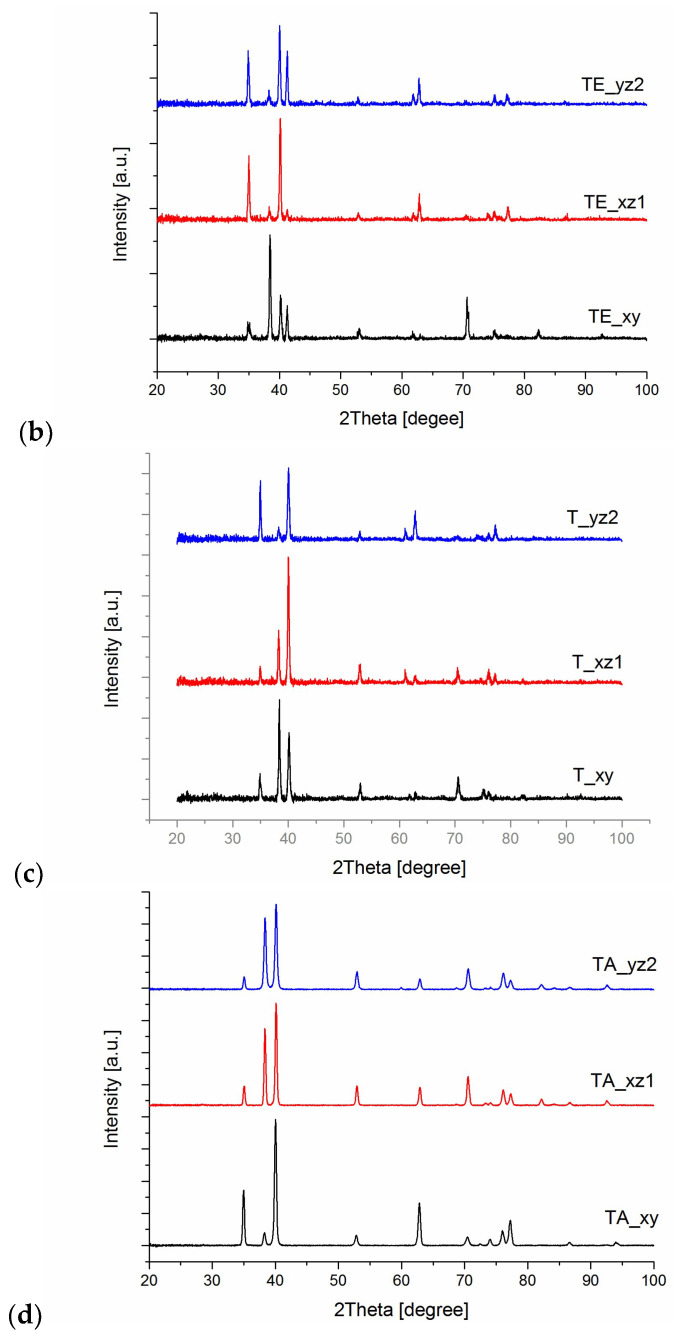
WAXS radial profiles of TE in comparison to T and TA: profile determined from the top surface for all samples (**a**); profiles determined from top, and two sides of the surfaces (s1, s2) for TA (**b**), T (**c**), TA (**d**).

Pure titanium crystal growth and phases are determined according to the parameters of the manufacturing process. During crystal growth, different kinds of dislocations or defects can be formed. Therefore, titanium crystal may contain defects such as dislocations cells and walls, statistically stored random dislocation, geometrically necessary dislocations, disclinations or disconnections, dislocation loops or a combination of these defects [25]. The WAXS profiles of the XY, XZ1, YZ2 cubic surfaces of explosive deformed titanium TE, initial state pure titanium T and titanium alloy TA are illustrated in Figure 4; the diffraction profiles of T and TE might be indexed as a hexagonal α-Ti type structure (the space group of P63/mmc). The TE profile exhibits additional peaks that are characteristic of the ω phase. The martensitic phase with (11 2 0) was observed on all of the analyzed surfaces (XY, XZ1, YZ2) of TE. Previously, after explosive deformation treatment, a peak of the ω phase orientation was observed within 1400 μm from the treated surface [26]. In our case, the profiles suggest the occurrence of explosive-induced α-ω martensitic transformation in the whole volume. The direct mechanism of the pressure driven α→ω martensitic transformation in pure titanium has been described by several authors [27]. The WAXS profiles of T and TE indicate diffraction from a crystal structure with maximums at 35°, 38.5°, 40.3° and 53.1° corresponding to the (100), (002), (101) and (102) lattice planes. The TA profiles are characteristic of the β-phase (bcc), as reported by other authors [28,29]. Comparing the analysis direction of the same sample (XY, XZ1, YZ2), a change in the relative intensities was observed for the main peaks: (100), (002) and (101). It is well-known that for an ideal perfect crystal, the scattering from atoms adds to the construction under Bragg’s conditions, which results in a delta function (δ-function)-like intensity distribution. Defects present in crystals may break the long range symmetry of the crystal lattice, which redistributes the scattering intensity. Point defects, small dislocation loops and precipitates undergo a change in their integral intensity, a shift in their peak position and diffuse scattering while maintaining the symmetry of the line shape [30]. Defects of the second kind cause asymmetrical peak broadening; the second kind includes dislocations, disclinations/disconnections, disclination dipoles, stacking faults, twins, boundaries, long cylinder-like chains of impurities, etc.

A strong anisotropic structure is clearly observed in the WAXS diffraction profiles of the TE samples cut perpendicular and parallel to the explosively hardened surface. The ratio of the integral intensity of plane (100) to plane (002) is 0.1 for perpendicular to the XY, 7 to XZ1 and 6.3 YZ2, respectively (Figure 4).

### 4.2. Mechanical Properties—Low Cycle Fatigue Test

**Figure 5:**
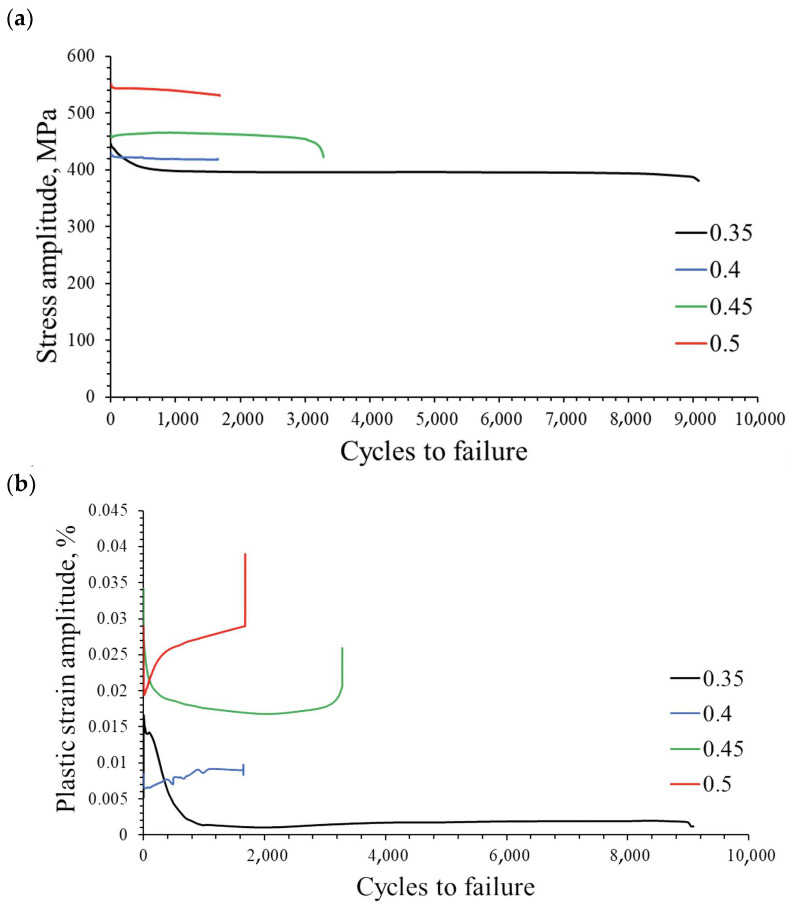
Stress amplitude (**a**) and plastic strain amplitude (**b**), as a function of the number of cycles.

A low-cycle fatigue test at total strain amplitudes of 0.35%, 0.4%, 0.45% and 0.5% allowed us to observe how the stress amplitude and plastic strain amplitude change as a function of the number of cycles (Figure 5a,b).

**Figure 6:**
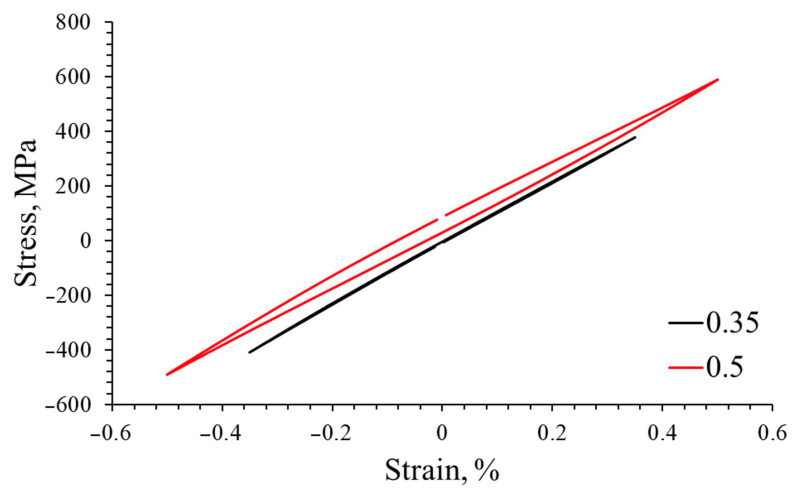
Hysteresis loops for strain amplitudes of 0.35% and 0.5%.

The comparison of the stabilized hysteresis loops for strain amplitudes of 0.35% and 0.5% indicates an increase in energy dispersion in the cyclically deformed material at higher strain amplitudes (Figure 6).

A graph of the cyclic strengthening is shown below.

**Figure 7:**
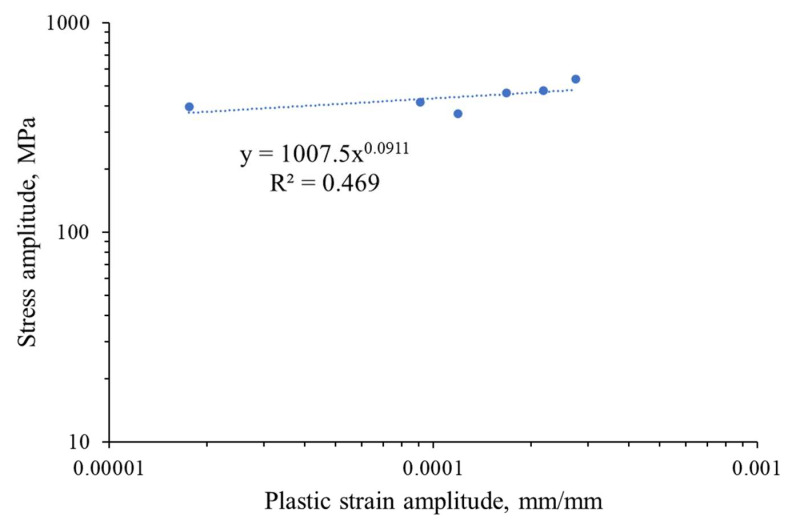
Strengthening grade 2 titanium for the stress–strain relationship during fatigue testing.

The resulting curve can be described by the equation:

*σ_a_* = *K*′(*ε_ap_*)*^n^*^′^ (1)

where *K*′ is the cyclic amplification factor and *n*′ is the exponent of cyclic amplification. The plastic strain amplitude and stress amplitude are not constant during fatigue testing; therefore, only their stabilized values, which are taken into consideration, allow us to describe the cyclic properties in most of the low-cycle fatigue life. The discussed equation connects the plastic strain and stress amplitude for the stabilized state based on the Ramberg–Osgood relationship, and the established parameters are considered to be constant for the tested material. The values of the equation are shown in the graph (Figure 7) and in Table 2.

### 4.3. Mechanical Properties—Residual Stresses

**Figure 8:**
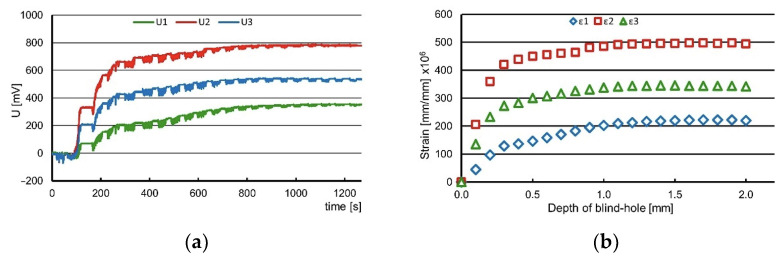
The changes in voltage recorded during one of the measurements (**a**) and the computed strains for the same example (**b**), where 1, 2 and 3 refer to the number of rosette gauges.

The output data of this research were the changes in voltage as a function of the blind-hole depth recorded with the increment of 0.1 mm. The output voltage was transmitted from the rosette to the channels of the ESAM Traveler Plus strain gauge bridge, where it was amplified. An example of a graph showing the changes in voltage recorded during the test is presented in Figure 8a.

The calculation of the strain values was conducted by employing Equation (2).

Ε = 4 · U/(U_0_ · N · K · A) · 10^6^ [µε] (2)

where:

U—output voltage [V];
U_0_—supply voltage [V];
N—coefficient depending on the bridge type—for quarter-bridge N = 1;
K—strain gauge constant;
A—coefficient of output signal amplification.

The characteristics of the strain changes as a function of the blind-hole depth were obtained during the calculations (see Figure 8b). To determine the values of the principal stresses and their direction, the following equations were applied:

(3) $$\sigma_{max}=\frac{\varepsilon_{1}+\varepsilon_{2}}{4\cdot A}-\frac{1}{4\cdot B}\sqrt{{\left(\varepsilon_{3}-\varepsilon_{1}\right)}^{2}+{\left(\varepsilon_{3}+\varepsilon_{1}-2\varepsilon_{2}\right)}^{2}\;}\left[\mathrm{MPa}\right]$$

(4) $$\sigma_{min}=\frac{\varepsilon_{1}+\varepsilon_{2}}{4\cdot A}+\frac{1}{4\cdot B}\sqrt{{\left(\varepsilon_{3}-\varepsilon_{1}\right)}^{2}+{\left(\varepsilon_{3}+\varepsilon_{1}-2\varepsilon_{2}\right)}^{2}\;}\left[\mathrm{MPa}\right]$$

(5) $$\alpha =\frac{1}{2}arctg\frac{\varepsilon_{1}-2\varepsilon_{2}+\varepsilon_{2}}{\varepsilon_{2}-\varepsilon_{1}}\left[\mathrm{MPa}\right]$$

where:

*σ_max_*, *σ_min_*—principal stresses;
*ε*_1_, *ε*_2_, *ε*_3_—strains measured on strain gauges number 1, 2 and 3;
*A*, *B*—coefficients depending on material properties and geometry of rosette and hole;
*α*—angle between strain gauge no. 1 and the direction of the nearest principal stress.

The calculations were carried out assuming a constant value of Young’s modulus of 103 GPa and Poisson’s ratio of 0.34. The determination of the stress value and its character was accomplished using the software H-Drill (http://www.schajer.org/, accessed on 13 November 2023), where the input data were the strains established in accordance with Equation (2), Young’s modulus, Poisson’s ratio, hole diameter and type of rosette. The obtained values of the principal residual stresses are presented in Table 3. The measurements were conducted at six points of the examined plate. The data calculated at three depths are presented because the residual stresses in the analyzed case are highly variable. The second depth is irregular and was 0.5 mm, 0.7 mm or 1 mm if the most significant stress change was observed at this depth.

**Figure 9:**
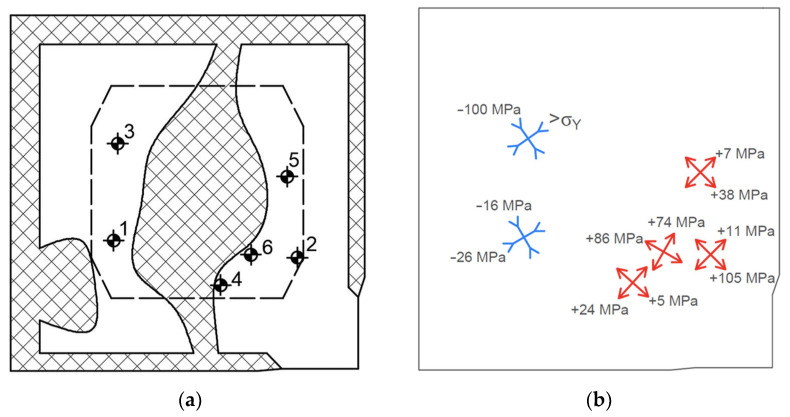
The location of the measurement points (**a**) and determined near-surface residual stresses with their orientation (**b**) where: blue—compressive stress; red—tensile stress.

The location of the measurement points is shown in Figure 9a, where the hatched field is the zone in which measurements cannot be made (see Figure 3). The dashed line limited the field where measurements could be carried out due to the capabilities of the RS-200 system (see Figure 2).

Although the measurements were made to the depth of 2.0 mm, residual stresses can only be determined to the maximum depth of 1.0 mm. This is the limitation of the method used in the case of nonuniform stresses being present. In addition, the residual stresses were determined in plate T at the initial state (not affected by the shock wave), where near-surface *σ_max_* residual stresses ranging between −170 MPa and −420 MPa at the depth of up to 0.4 mm were observed. At a depth of over 0.4 mm, their value stabilized at the level of −40 MPa to +70 MPa. The values and character of the residual stresses in TE are very heterogeneous, both when considering them in one plane—e.g., near-surface (see Figure 9b)—and when analyzing the depth of the material. This is caused not only by the dynamics of the processing, but also by the conditions of its realization, e.g., the influence of SUP. It can be seen that the residual stresses close to the place of detonation initiation are tensile, and behind the SCZ, they are compressive, even exceeding the yield stress (point no. 3). Moreover, the orientation of the principal stresses is connected with the direction of the detonation wave propagation. At all of the measuring points, near-surface layers with different thicknesses—ranging between 0.2 mm and 0.8 mm—were detected, which characterized the other state of the residual stresses and mechanical properties; this layer was much more difficult to drill. The residual stresses stabilized below this layer, which can be observed by analyzing the values of the residual stresses determined at depth 1.0 mm, but especially by analyzing the individual changes in the stresses established for each measurement, because the stresses stabilized at different values. A similar change in the residual stresses is observed in the material at the delivery state. When considering the above data, it is important to remember the most important factor; namely, that the calculations were carried out with a single value of Young’s modulus and Poisson’s ratio. After explosive processing, the material possesses other structure and mechanical properties; thus, this can significantly affect the results, although the magnitude of this impact is difficult to quantify. The effect of the described factors could be, among others, the theoretical exceeding of the yield point at point no. 3, which results from the limitation of the method used, to the range of elastic deformation. The actual strength properties of the material in the analyzed area could be different from the assumed ones.

### 4.4. Corrosion

Corrosion tests were carried out, based on which the corrosion potential and corrosion current density were identified. The characteristics of the material’s behavior at variable electrochemical potentials were determined. The values of the designated numerical data are presented in Table 4.

It has been observed that the transition to the passive state occurs almost instantaneously. The values determined using direct current electrochemical tests can be described as being similar. The current densities are minimal and do not vary significantly, while the annual corrosion rates are negligible. The explosively hardened sample is characterized by greater speed and intensity of the corrosion processes. However, the change in parameters is negligible and does not exceed the corrosion rate on a scale of 1 µm per year.

### 4.5. Cytotoxicity

**Figure 10:**
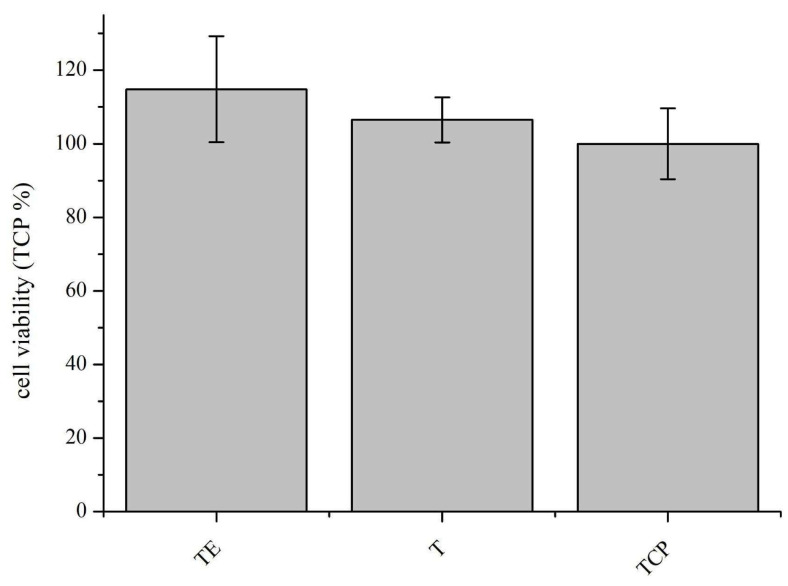
Results of a 24-h cytotoxicity study using extracts from pure titanium materials.

The cytotoxicity study on pure titanium materials after explosive deformation (TE) performed in relation to the control material—pure titanium without explosive deformation (T)—showed the lack of cytotoxicity of both materials. In comparison to the pure culture medium (TCP), the control showed slight differences in favor of both titanium materials; however, they are not statistically significant, as can be seen in the graph below (Figure 10). Error bars represent standard deviation values. Cytotoxicity, according to ISO 10993-5 [31], is stated for materials for which the result of the viability test is less than 70% of the value obtained for the control sample. The in-vitro test shows the lack of cytotoxicity of pure titanium after explosive deformation.

### 4.6. Adhesion, Spreading and Proliferation of Osteoblasts

**Figure 11:**
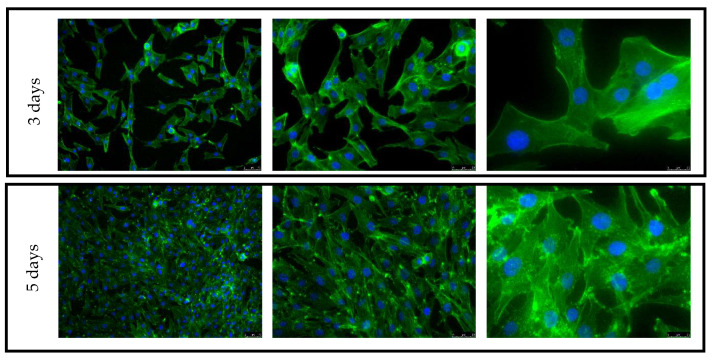
Fluorescence microscopy images of MG63 cultured on TCP for 3 and 5 days.

**Figure 12:**
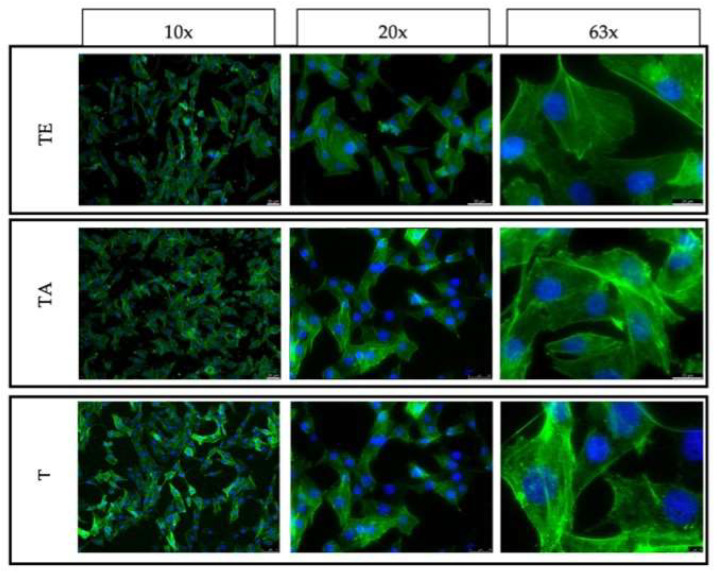
Fluorescence microscopy images of MG63 cultured on the top surfaces of different titanium samples for 3 days: XY surface.

**Figure 13:**
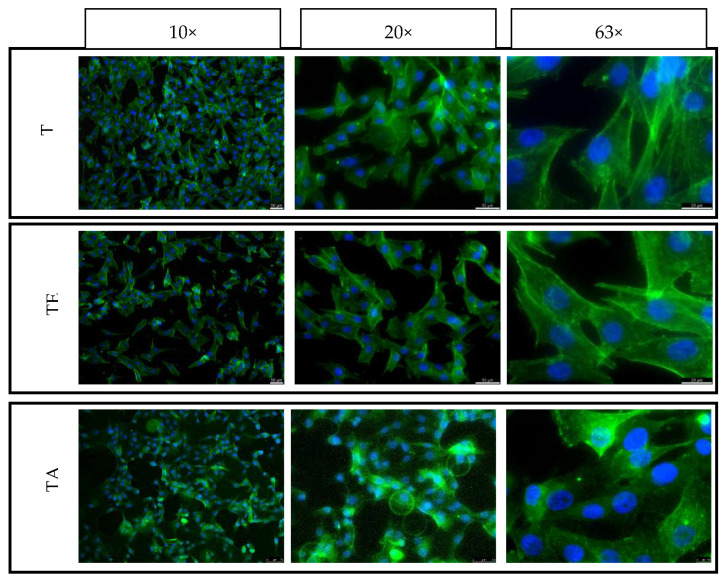
Fluorescence microscopy images of MG63 cultured on the side surfaces of different titanium samples for 3 days: XY1 surface.

**Figure 14:**
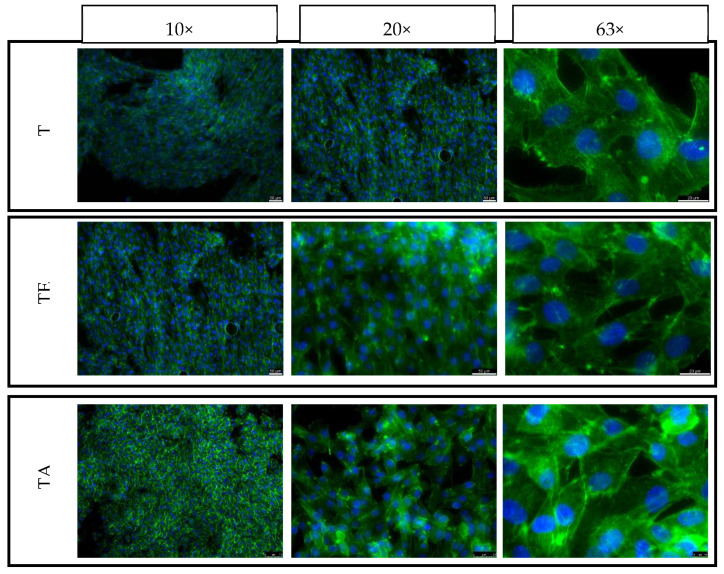
Fluorescence microscopy images of MG63 cultured on the top surfaces of different titanium samples for 5 days: XY surface.

**Figure 15:**
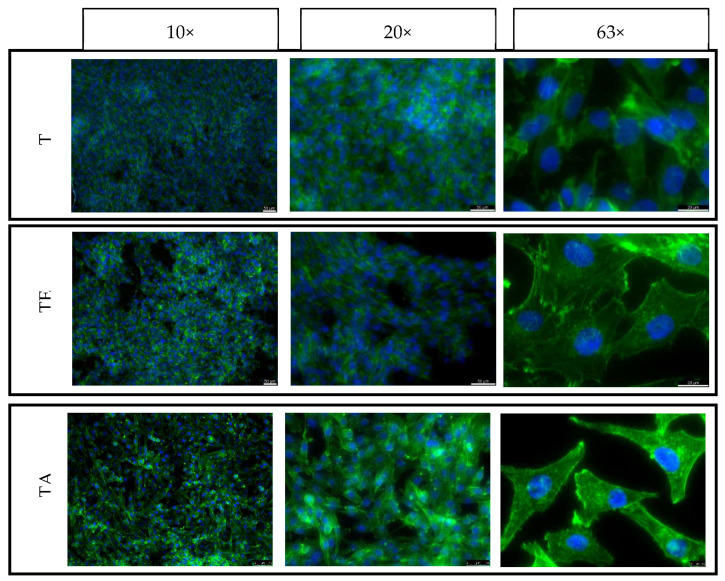
Fluorescence microscopy images of MG63 cultured on the side surfaces of different titanium samples for 5 days: XY1 surface.

The control consisted of cells cultured on TCP is shown in Figure 11. Cells growing on both the upper and side surfaces were assessed after 3 and 5 days of culture for both types of pure titanium (T)—after explosive deformation (TE) and in the initial state)—together with the titanium alloy material (TA) (Figure 12, Figure 13, Figure 14 and Figure 15). The cell morphology observed in the recorded images proves the correct growth and proliferation of the MG63 cell line. With the exception of minor differences in the arrangement of the cells on the surface of the materials, it is difficult to find features that indicate an abnormal morphology of the cells growing on all of the types of materials and on both surfaces.

The images with the highest magnification clearly show oval, correct-shaped cell nuclei and an extensive actin skeleton with numerous places of attachment to the surface and clearly visible individual filopodia. This proves that the surface of the material is well tolerated by cells, they willingly attach to it and it is an attractive substrate for their growth. The similar morphology of the cells on the surfaces of all types of titanium means that the deformation of titanium by means of an explosion has no negative effect on the cellular response of the MG63 line in direct contact. In addition, no clear differences were observed in the results for the XY and XZ1 surfaces for both time points, which for the material after explosive deformation, indicates no changes in the properties significant for cell cultures, regardless of the direction of the deformation force.

### 4.7. ECM Secretion Tests in Direct Contact

**Figure 16:**
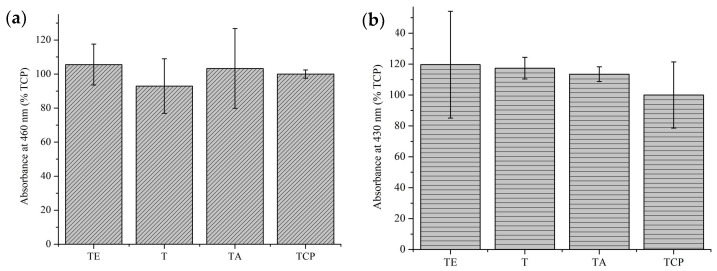
(**a**) collagen deposition after 7 days of MG63 culture in direct contact, analyzed using Direct Red 80 assay; (**b**) show Ca^2+^ deposition after 7 days of MG63 culture in direct contact determined by the results of the Alizarin Red assay.

The results of both of the performed tests, Direct Red 80 and Alizarin Red, are important from the functional point of view of titanium, i.e., in bone implants. One of the methods for assessing the biofunctionality of titanium materials intended for the stabilization of bone fractures is to determine the cellular response to them in-vitro. A good indicator of how cells respond to the surface of such a material is taking a closer look at the extracellular matrix they produce, or more precisely, its ingredients, including determining the amount of protein—collagen, as well as bone mineral component—calcium ions. As collagen is the primary protein in the composition of bone, assessing how much collagen an osteoblast culture secrets in direct contact with a material is essential to test whether this material is suitable to be used for bone regeneration implants. The results of the Direct Red 80 assay (Figure 16a) show that all of the tested titanium materials had a similar influence on the MG63 osteoblast culture in the amount of collagen that was produced over 7 days of the experiment.

Similarly, the Alizarin Red staining results proved that the osteoblasts cultured on the surface of all the titanium samples secreted Ca^2+^ in the same or slightly greater amount compared to the control TCP assay (Figure 16b).

## 5. Conclusions

On the basis of the obtained results, the following conclusions can be stated:

1. Phase structure analyses via WAXS confirmed that explosive-induced α phase-ω martensitic transformation occurred in the whole volume of the titanium after explosive deformation. Additionally, crystallographic anisotropy was observed in all of the analyzed samples.
2. Residual stresses are very non-uniform, both when considering the near-surface layer or deep into the material. The presence of a layer with a different, strongly changing distribution of residual stresses—the thickness of which ranges between 0.2 mm and 0.8 mm—was found. Below this layer, the state of residual stresses stabilizes.
3. The corrosion tests allow us to conclude that, following deformation by explosion, the corrosion properties do not change significantly.
4. The titanium deformed by explosion indicates similar biocompatibility as pure non-deformed titanium and is commonly implanted in titanium ally (type). The phase composition and crystallographic orientation do not influence the morphology of bone cells.

## Figures and Tables

**Table 1:** Ringer’s solution used for corrosion tests.

Substance	Contents [g/dm^3^]
NaCl	6.0
KCl	0.075
CaCl_2_	0.1
NaHCO_3_	0.1

**Table 2:** The established parameters of the cyclic stress–strain plot.

Cyclic Strength Coefficient, *K*′ [MPa]	Cyclic Strain Hardening Exponent, *n*′	Coefficient of Determination, R^2^
1007.5	0.0911	0.469

**Table 3:** The results of residual stresses measurements of TE and in the delivery state.

	Number of measuring point								
	# 1			# 2			# 3		
depth [mm]	0.0	0.5	1.0	0.0	0.5	1.0	0.0	0.2	1.0
	residual stresses *σ* [MPa] and angular orientation α [°]								
*σ_max_*	−16	−1	25	105	76	47	−100	26	4
*σ_min_*	−26	−34	−51	11	10	7	>*σ_Y_*	−74	−124
	Number of measuring point								
	# 4			# 5			# 6		
depth [mm]	0.0	0.3	1.0	0.0	0.7	1.0	0.0	0.6	1.0
	residual stresses *σ* [MPa] and angular orientation α [°]								
*σ_max_*	24	−186	59	38	71	198	86	42	35
*σ_min_*	5	−292	−7	7	−19	96	74	−23	−11

**Table 4:** Values of potentials and corrosion current densities for the tested samples and the value of the corrosion rate given in mm/year.

Material	U [mV]	I [nA/cm^2^]	Corrosion Rate (CR) [mm/year]
T (pure titanium non deformed	−469	4.6	0.00008
TE (pure titanium deformed)	−490	5.5	0.0001

## Data Availability

Data are contained within the article.
